# Sea cucumber sulfated polysaccharides extract potentiates the anticancer effect of 5- fluorouracil on hepatocellular carcinoma cells

**DOI:** 10.1038/s41598-025-06496-7

**Published:** 2025-06-23

**Authors:** Samah E. Ismail, Neveen A. Hussein, Mona M. Rashad, Amany M. El-Sikaily, Abd El-Latif A. Hassanin, Esmail M. El-Fakharany

**Affiliations:** 1 Applied Medical Chemistry Department, Medical Research Institute, Alexandria University, Alexandria, Egypt; 2 Marine Pollution Department, National Institute of Oceanography and Fisheries, Ministry of Higher Education and Scientific Research, Alexandria, Egypt; 3 Marine Biotechnology Department, National Institute of Oceanography and Fisheries, Ministry of Higher Education and Scientific Research, Alexandria, Egypt; 4 Protein Research Department, Genetic Engineering and Biotechnology Research Institute, City for Scientific Research and Technology Applications, Alexandria, Egypt; 5 Pharmaceutical and Fermentation Industries Development Center, City of Scientific Research and Technological Applications (SRTA-City), New Borg EL-Arab, Alexandria, 21934 Egypt

**Keywords:** Sea cucumber, Sulphated polysaccharides, Angiogenesis, Apoptosis, Hepatocellular carcinoma, Biochemistry, Cancer, Cell biology, Drug discovery, Molecular biology

## Abstract

Sea cucumber represents a potential marine source of high value compounds with medicinal properties especially its anti-cancer activity. Sea cucumbers contain numerous biomolecules, including sulfated polysaccharides (Ps) which have enormous therapeutic and nutraceutical potential. This study aimed to investigate anticancer effect of Ps extracted from sea cucumbers on hepatocellular carcinoma. This study was in vitro study conducted on HepG-2 cells and normal wish cells that were divided into four groups: Group I including untreated cells, Group II including cells treated with different concentrations of 5-FU, Group III including cells treated with various concentrations of Ps extract. Group IV including cells treated with different concentrations of combined 5-FU and Ps extract. The extracted Ps were characterized using FT-IR, HPLC, and GC–MS. The anticancer effect of Ps extract was determined using cytotoxicity MTT assay, DNA fragmentation assay, wound healing assay, colony formation and soft agar assay. Also, the effect of Ps extract on *VEGF*, *survivin*, *BAX* and *BID* gene expression was determined by qRT-PCR and its effect on Bcl2 and BAK protein level was determined by western blotting technique. The results indicated that sea cucumber Ps extract either alone or in combination with 5-FU reduced HepG-2 and wish cell viability with higher selectivity index. Also, it inhibited both adherent and non-adherent colony forming ability and cell migration of HepG-2 cells. Moreover, it was significantly downregulated *VEGF*, *survivin* and Bcl2 while, it was significantly upregulated *BAX*, BAK and *BID*. In conclusion, sea cucumber Ps extract may be an effective chemotherapeutic agent against HCC.

## Introduction

Primary liver cancer is the sixth most common diagnosed cancer and the third leading cause of cancer-related mortality globally in 2022. Hepatocellular carcinoma (HCC) accounts for 75% to 85% of primary liver cancer cases^1^. In order to acquire a sufficient blood supply for tumor growth, HCC, a highly vascularized tumor, produces large amounts of vascular endothelial growth factor (VEGF) to form many blood vessels. Thus, there is a correlation between tumor angiogenesis and HCC progression^2^.. Angiogenesis has been implicated in cancer growth owing to tumour’s ability to release chemical signals that initiate mitogenic and antiapoptotic signaling pathways and to facilitate metastatic spread of cancer cells through haematogenic and lymphogenic diffusion^3^.

Apoptosis is one of the primary changes that controls the progression of cancer^4^. Apoptosis, also known as programmed cell death, is defined by specific morphological alterations and biochemical processes that depend on energy. This process is essential for fetal development and maintaining tissue homeostasis in multicellular organisms^5^.. The mechanism of apoptosis is complex and involves numerous pathways. Defects in apoptosis pathways may lead to malignant transformation of the affected cells, tumour metastasis and resistance to anticancer drugs. as apoptosis is a popular target of many treatment strategies, it plays an important role in the treatment of cancer^6^.

Survivin is a multifunctional protein that plays a crucial role in mitosis and inhibits apoptosis^7^. B-cell lymphoma 2 (Bcl2)-associated X protein (BAX) and Bcl2 antagonist killer 1 (BAK) are essential to the mitochondrial pathway of apoptosis. Their activation during apoptosis involves several conformational changes, which are accompanied by their homo-oligomerization within the mitochondrial inner membrane^8^. Furthermore, upregulation of Bcl2 gene expression enhances cell survival through its anti-apoptotic activities. Bcl2 contains BH4 and transmembrane domains, which enable it to anchor to cellular membranes and influence mitochondrial membrane permeability. This action allows Bcl2 family proteins to participate in the regulation of apoptosis by counteracting pro-apoptotic signals^9^.

BH3-only proteins, including Bcl2 interacting mediator of cell death (BIM) and BH3 interacting-domain death agonist (BID), activate and oligomerize BAX and BAK, thereby promoting the continuation of the intrinsic apoptotic pathway. This activation drives the progression of apoptosis through both the mitochondrial and caspase-dependent pathways^10^.

Liver cancer is mostly treated with a number of methods, including radiofrequency, chemotherapy, targeted molecular therapy, liver transplantation, and surgical resection. But there are still significant problems with illness recurrence and drug toxicity or ineffectiveness, which have a detrimental effect on the patient’s quality of life following treatments^11^.. Thus, new treatments with high efficacy and minimal side effects are therefore desperately needed.

Natural compounds found in marine species can provide chemicals with medicinal, pharmacological, and nutritional uses^12^. Sea cucumbers play a crucial role in marine ecosystems and are commonly found in shallow waters with coral, rocks, or seaweed. They are known for their rich nutritional content and contain various active components, including bioactive peptides, as well as agents with anti-inflammatory, antioxidant, antimicrobial, anti-angiogenic, and anticancer properties^13^. A sulfated polysaccharide is the critical nutrient in sea cucumber, and it consists of two sulphated polysaccharides, namely fucosylated chondroitin sulphate and fucoidan sulphate^14,15^. A sulfated polysaccharide has significant physiological effects such as anticancer^16^, anti-bacterial activity^17^, hypolipidemic^18^, immune regulation^19^ and anti-inflammation^20^. However, the underlying mechanism of its effects is still unclear. Therefore, this study aimed to investigate the anticancer effect of sulfated polysaccharides extracted from sea cucumbers on hepatocellular carcinoma.

## Results

### Characterization of sea cucumber Ps extract

#### FT-IR

**Fig. 1:**
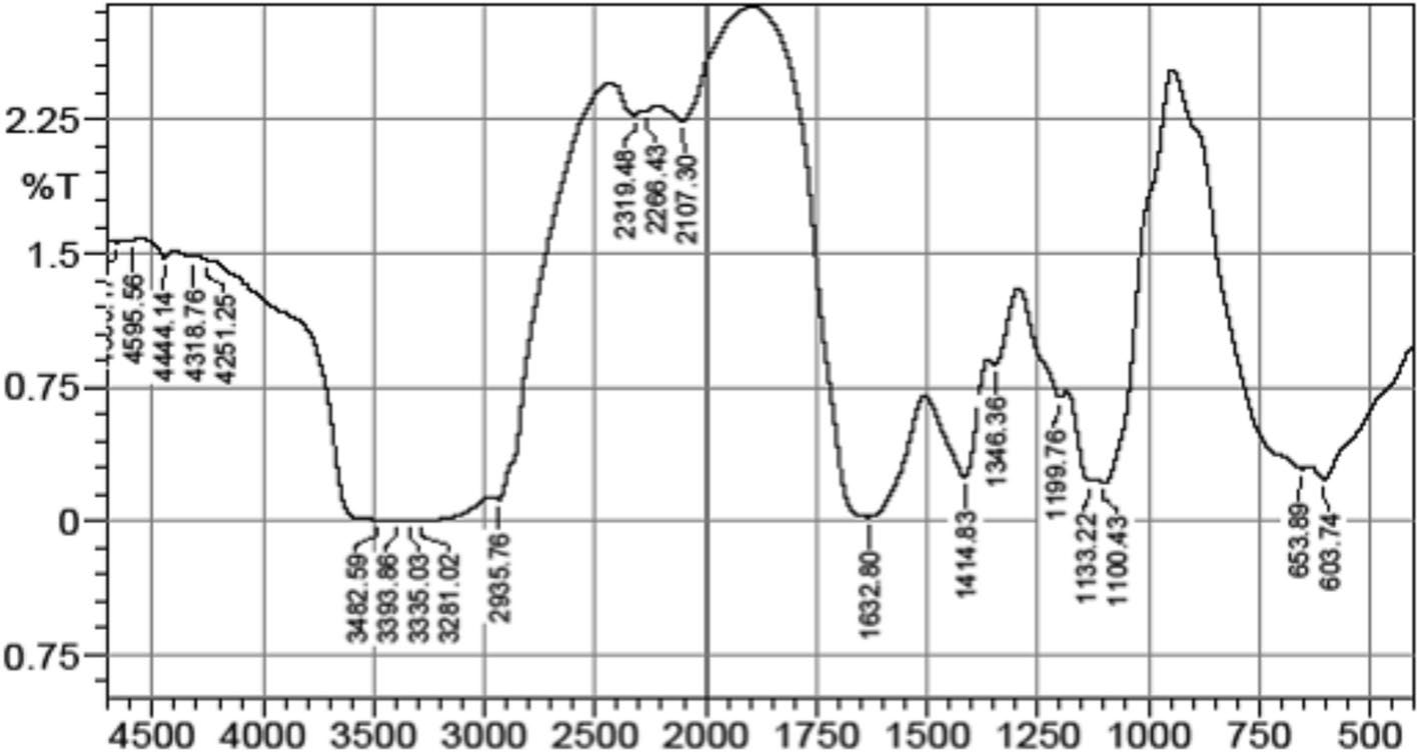
FT-IR chart of sea cucumber polysaccharide extract.

The most important frequencies are between 1,000 and 4,000 nm wave number (Fig. 1). The FT-IR spectra display the existence of sugar backbone (1,414–1,133 nm), and these spectra showed several vibration of sulfate (S = O) and bending vibration of sulfate (C-O-S) in axial position. The signals at 3,442 and 1,100 nm are from stretching vibration of O–H and C-O, respectively. A broad peak appear in the range of 3,423 nm is associated with O–H and/or N–H stretching resulting from the multiple intramolecular hydrogen bonding interactions. The presence of an aliphatic C-H stretching mode of the methylene and methyl group can be assigned at 2,935 nm. The absorption band at 1,632 nm is attributed to N–H bending vibration of amide. The absorption band at 1,133 nm is corresponded to C-N stretching of amino and/or amide entity. Also, secondary amine appears at 2,319 nm. Data obtained were provided in supplementary Table 1.

#### HPLC analysis

**Fig. 2:**
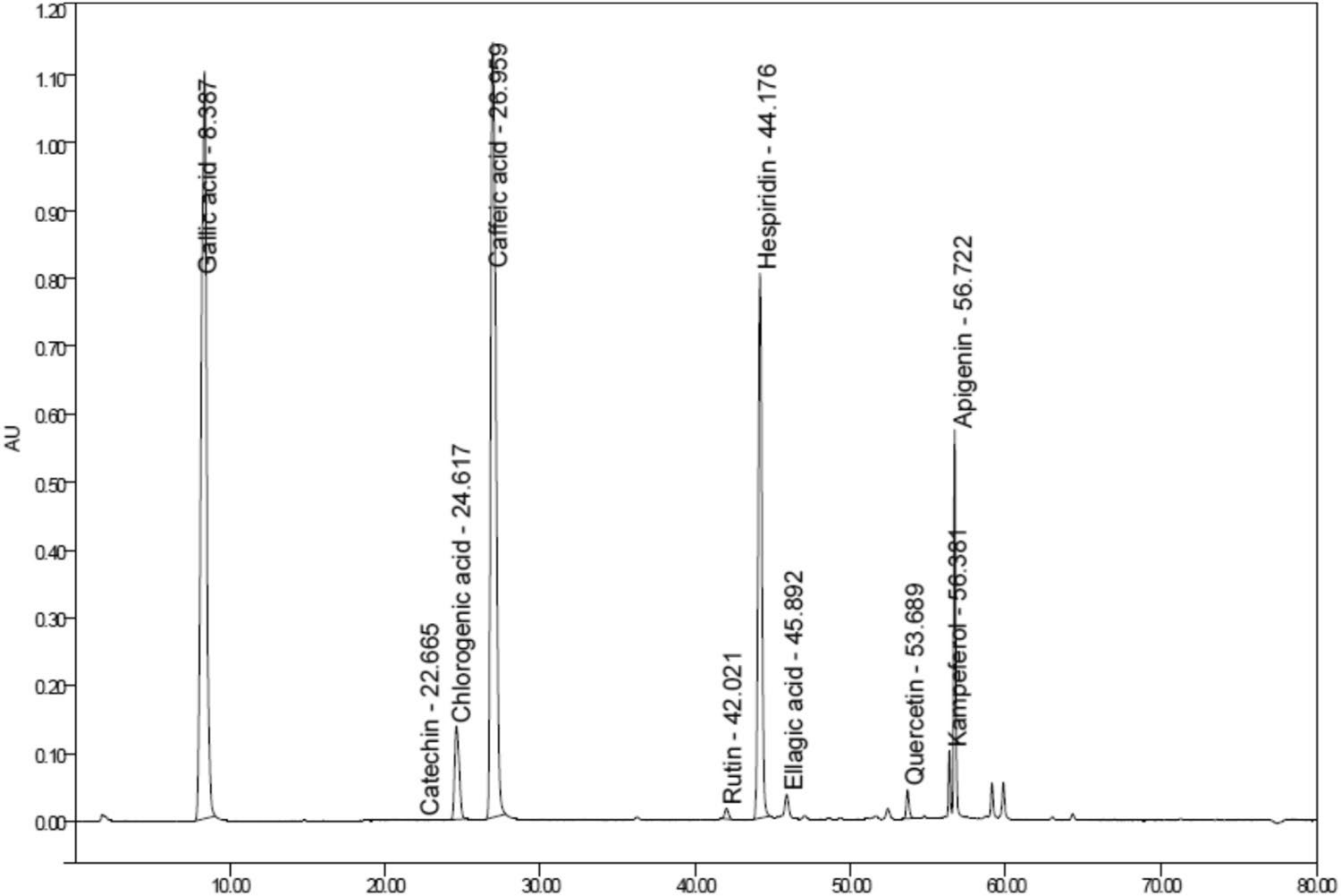
HPLC chromatogram of polysaccharide extract.

Characterization of the extracts by HPLC showed the existence of 10 phenolic and flavonoid compounds: gallic acid, catechin, chlorogenic acid, caffeic acid, rutin, hesperidin, ellagic acid, quercetin, kaempferol and apigenin detected. The major compounds are gallic acid (35.25%) and caffeic acid (34.84%), hesperidin (17.49%). Meanwhile, rutin recorded (0.36%). Also, ellagic acid, quercetin and kaempferol recorded (0.65, 0.68, 0.93%) respectively. This may be due to the varying level of sensitivity of some phenolic compounds towards applied temperature, vacuum and grinding (Fig. 2).

#### GC–MS analysis

**Fig. 3:**
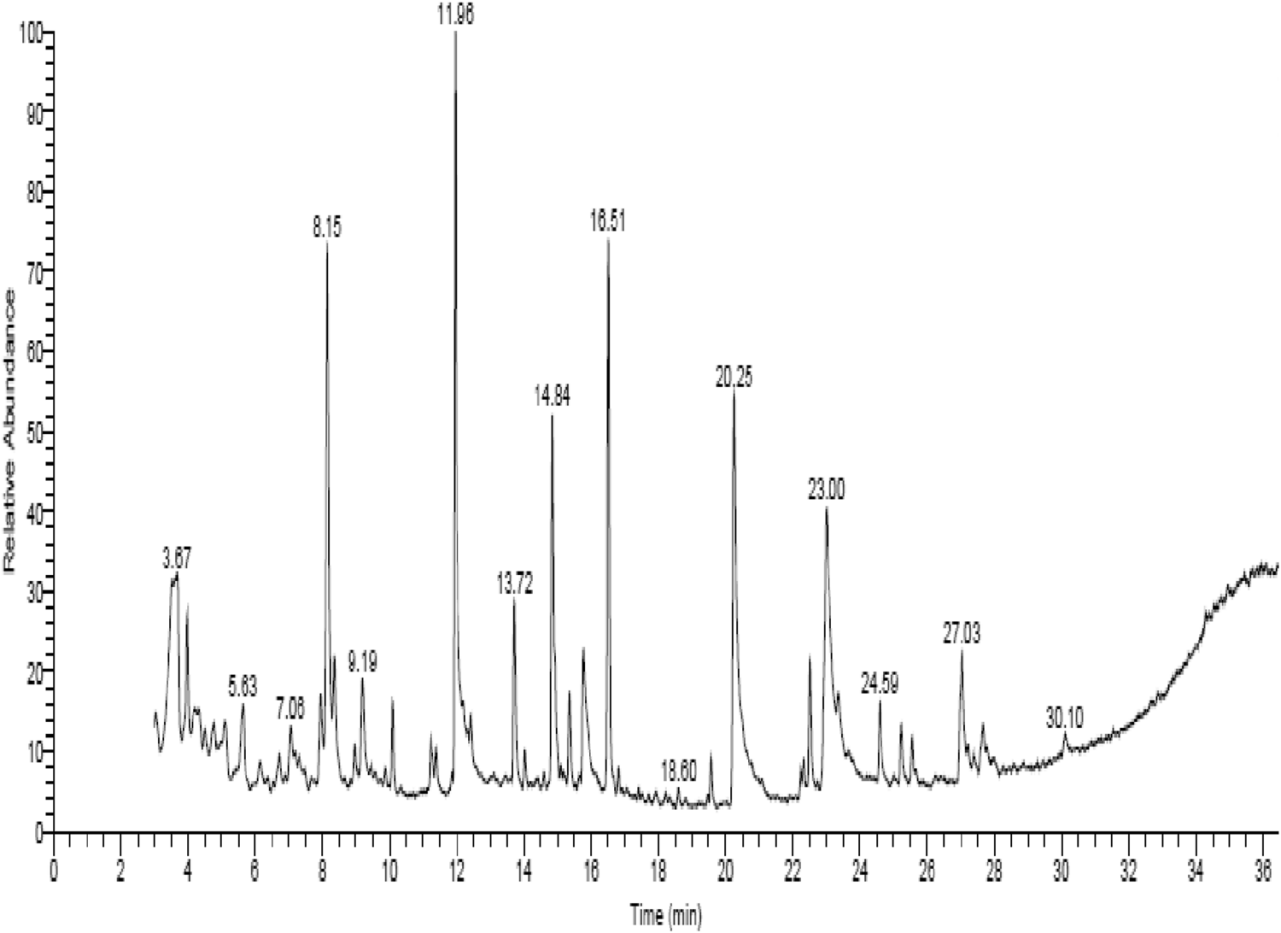
GC–MS chart of Sea cucumber Ps extract.

Gas chromatography mass spectrometry analysis revealed that composition of the most active ethanol Ps extract components were 32 compounds (Fig. 3). The nomenclature and chemical structure of bioactive compounds of sea cucumber Ps extract were provided in supplementary Table 2.

### The effect of 5-FU and/or Ps extract on the viability of wish cells and HepG-2 cells

**Fig. 4:**
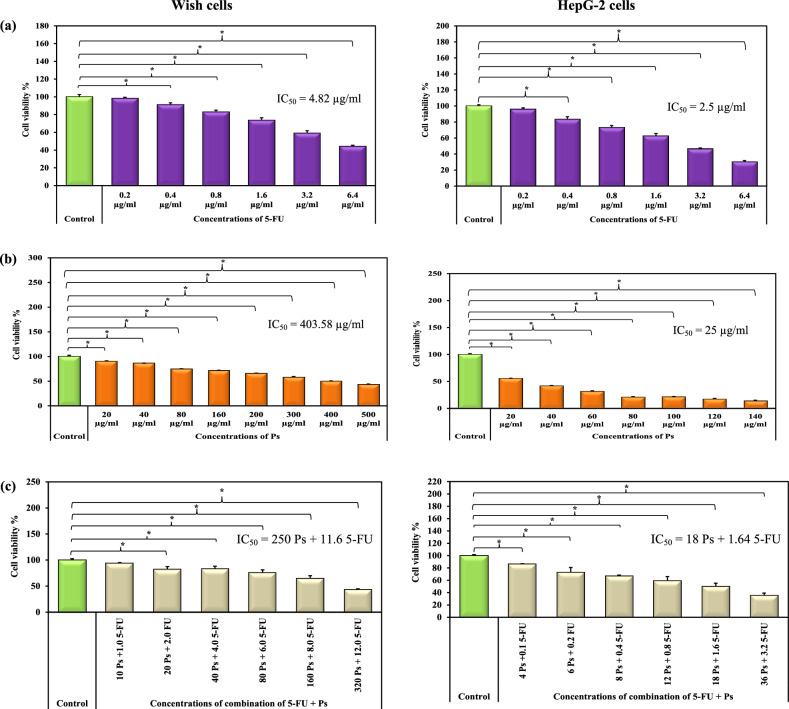
Bar chart representing the viability (%) of HepG-2 and Wish cells treated with different concentrations of 5-FU (**a**), Ps extract (**b**) and combination of 5-FU and Ps extract (**c**). *: Statistically significant at p ≤ 0.05.

Treatment of normal wish cells with different concentrations of 5-FU, Ps extract and combination of 5-FU and Ps extract significantly decreased cells viability in a concentration dependent manner (Fig. 4). The IC50 values of 5-FU was 4.82 µg/ml, Ps extract was 403.58 µg/ml and combination of 5-FU and Ps extract was 11.6 µg/ml 5-FU + 250 µg/ml Ps. Also, Treatment of HepG-2 cancer cells with different concentrations of 5-FU, Ps extract and combination of 5-FU and Ps extract significantly decreased cells viability in a concentration dependant manner. The IC50 values of 5-FU was 2.5 µg/ml, Ps extract was 25 µg/ml and combination of 5-FU and Ps extract was 1.64 µg/ml 5-FU + 18 µg/ml Ps (Figs. 7 – 9). Moreover, the results of the present study revealed that selectivity index of 5-FU was low 1.92 showing toxic effect on normal cells. While, SI for Ps extract was 16.14 and SI for 5-FU with Ps extract was 14.2.

### Morphological changes of HepG-2 cells

**Fig. 5:**
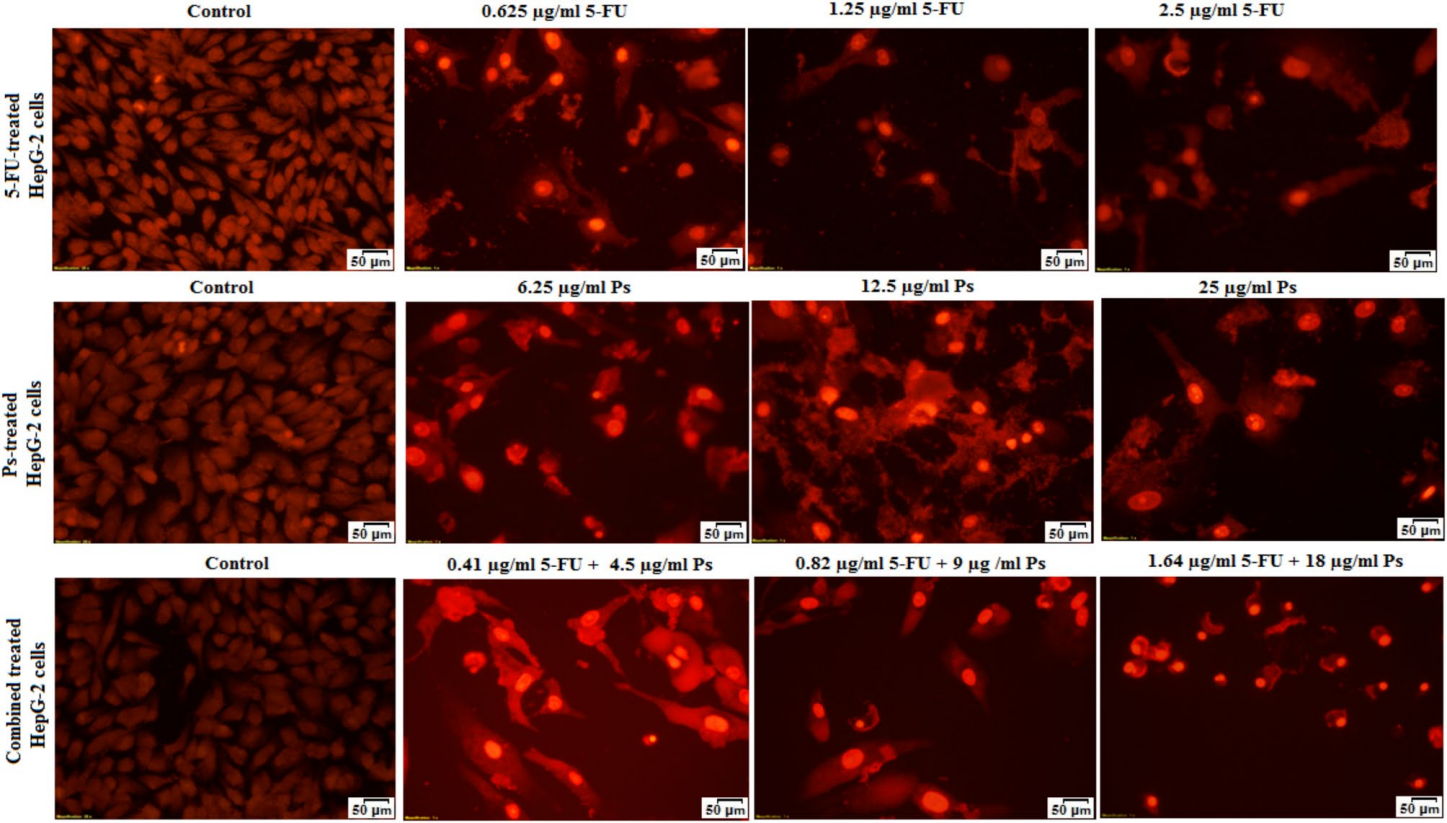
Investigation of the morphological changes of HepG-2 cells in all studied groups using single PI I staining of the treated HepG-2 cells.

Upon examining the morphological alterations in HepG-2 cells after 48 h of treatment with different concentrations of 5-FU, Ps extract and combination of 5-FU and Ps extract, it was observed that there were apoptotic changes in morphology of the treated-HepG-2 cells in a dose dependent manner (Fig. 5). It was observed that in 5-FU-treated group, HepG-2 cells showed fragmented nuclear bodies after 48 h of treatment with 0.625 μg/ml and showed some typical morphological characteristics of apoptosis, including the fragmented cell bodies and the plump nuclei after treatment of HepG-2 cells with 1.25 μg/ml of 5-FU when compared to untreated HepG-2 cells. Furthermore, treatment with high concentration of 5-FU (2.5 μg/ml) revealed destructed and dispersed cells with smaller size and denser cytoplasm compared to untreated cells.

Ps extract at low concentrations of 6.25 and 12.5 μg/ml showed a slight cytotoxicity with low morphological changes and fluorescence incidence inside the treated HepG-2 cells compared to untreated HepG-2 cells. Additionally, the Ps-treated HepG2 cells at concentration of 25 μg/ml showed a remarkable morphological alteration with apoptosis characteristics including shrunken cells and condensed cytoplasm, and the appearance of apoptotic bodies compared to untreated HepG-2 cells. On the other hand, it was observed that 4.5 μg/ml Ps + 0.41 μg/ml 5-FU and 9 μg/ml Ps + 0.82 μg/ml 5-FU showed fragmented HepG-2 cells and plump nuclei, which was similar with 1.25 μg/ml 5-FU treated HepG-2 cells. The treatment of HepG-2 cells with 18 μg/ml Ps + 1.64 μg/ml 5-FU combination showed small cell size (shrunken HepG-2 cells), broken nuclei, condensed chromatin and the appearance of apoptotic bodies which were important indicators of cell apoptosis compared to untreated cells.

### The effect of 5-FU and/or Ps extract on DNA fragmentation

**Fig. 6:**
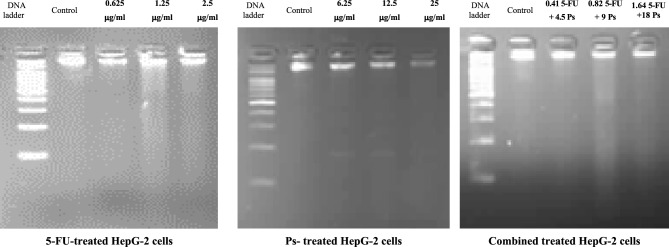
DNA fragmentation of HepG-2 cells in all studied groups.

The results of this study revealed that 5-FU, Ps extract and combination of 5-FU and Ps extract at low concentration induced only a minor increase in DNA fragments, whereas significant DNA fragmentation was observed after 48 h of treatment in a concentration dependent manner (Fig. 6).

### Anti-migration activity of 5-FU and/or Ps extract

**Fig. 7:**
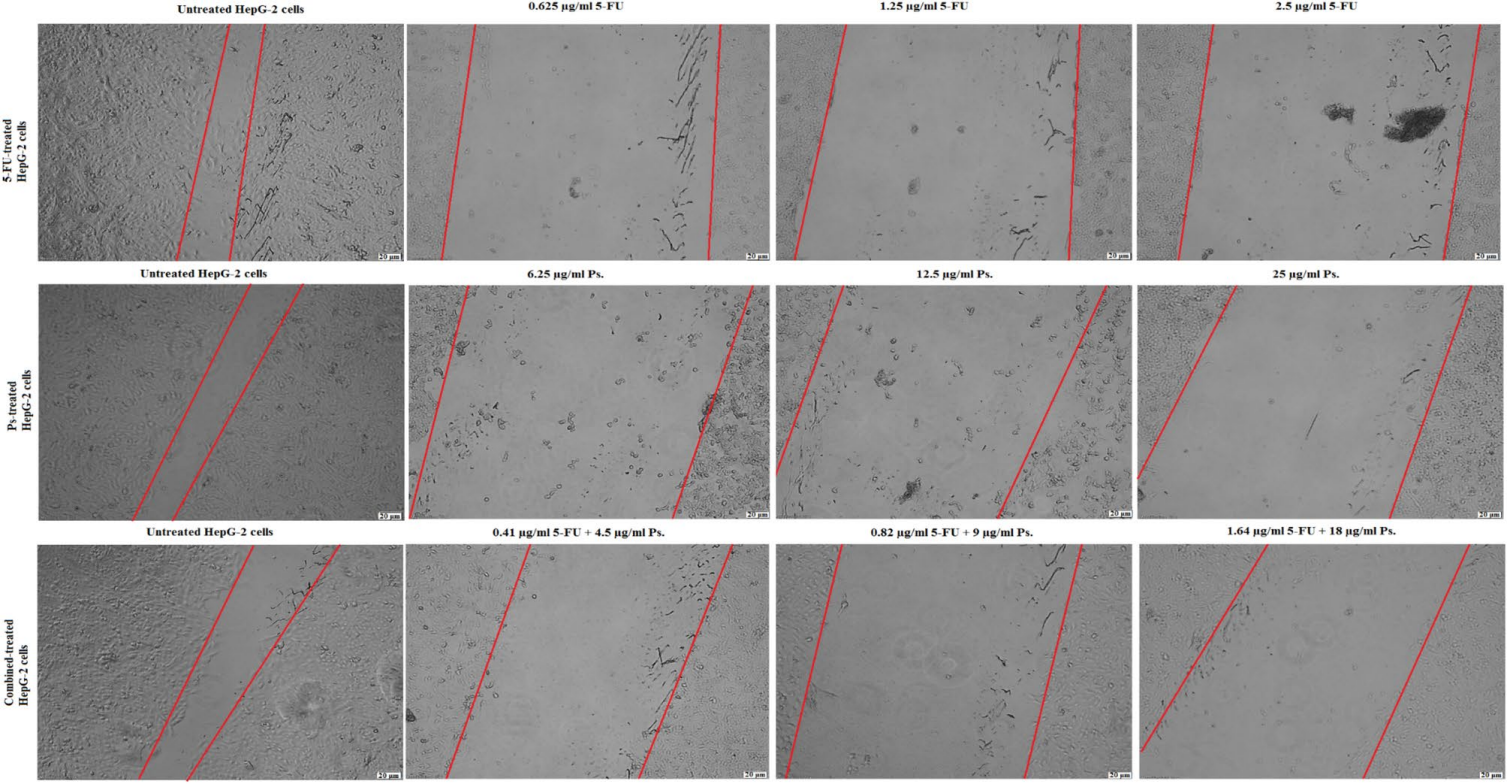
Wound healing of HepG-2 cells in all studied groups as observed at 48 h post-scratching after treatment with 5-FU, Ps and combination (5-FU + Ps). All experiments were performed in triplicate, and scale bar is 20 μm.

**Fig. 8:**
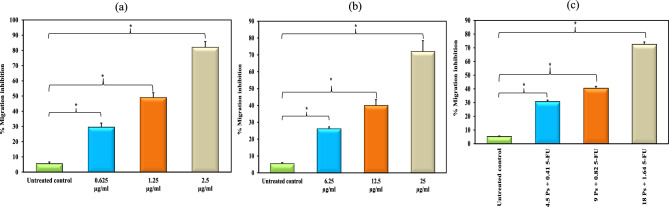
Bar chart representing migration inhibition (%) in HepG-2 cells treated with different concentrations of 5-FU (**a**), Ps extract (**b**) and combination of 5-FU and Ps extract (**c**). *: Statistically significant at p ≤ 0.05.

Treatment of HepG-2 cells with 5-FU, Ps extract and combination of 5-FU and Ps extract decreased their migration compared to untreated HepG-2 cells in a concentration dependent manner. So that 5-FU, Ps extract and combination of 5-FU and Ps extract had a high potency to inhibit migration activity (Figs. 7 & 8).

### The effect of 5-FU and/or Ps extract on adherent colony formation

**Fig. 9:**
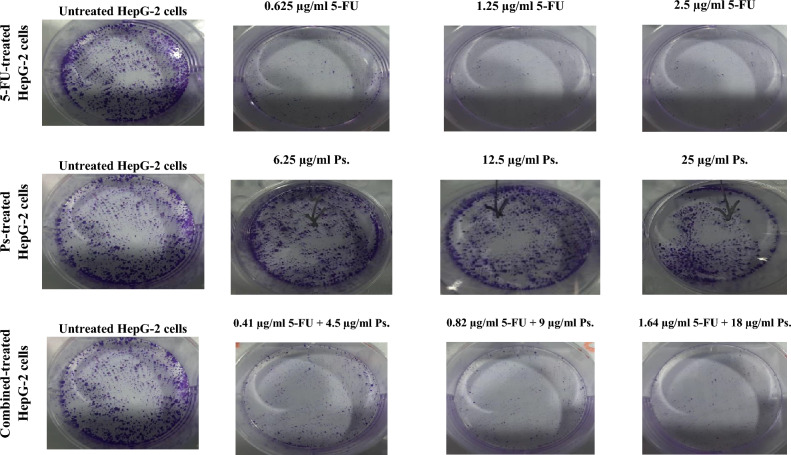
Colony formation for HepG-2 cells after 48 h treatment with different concentrations of 5-FU, Ps and combined 5-FU + Ps.

The results of the present study revealed that there was a decrease in number of colonies after 48 h treatment of HepG-2 cells with different concentrations of 5-FU, Ps extract and combination of 5-FU and Ps extract. The PE (%) was 25, 17 and 10% in cells treated with different concentrations of 5-FU (0.625, 1.25 and 2.5 µg/ml, respectively), while it was 90, 83 and 58% in cells treated with different concentrations of Ps extract (6.25, 12.5 and 25 µg/ml, respectively) and it was 42, 32 and 30% in cells treated with different concentrations of the combined drugs (5-FU and Ps) (0.41 5-FU + 4.5 Ps, 0.82 5-FU + 9 Ps and 1.64 µg/ml 5-FU + 18 µg/ml Ps, respectively) compared to untreated cells (Fig. 9).

### The effect of 5-FU and/or Ps extract on non-adherent colony formation

**Fig. 10:**
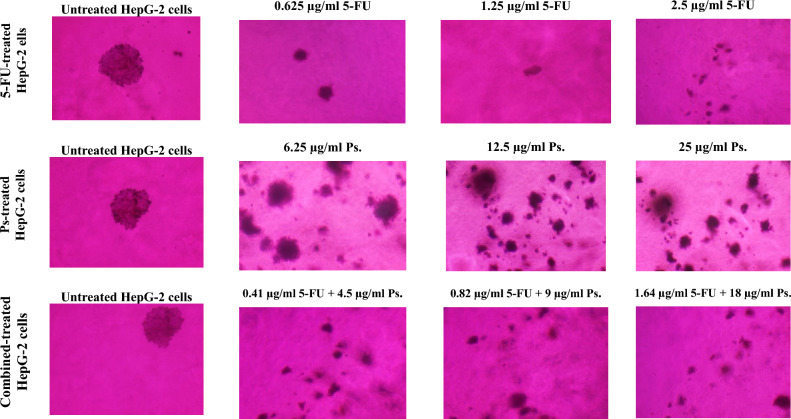
Soft agar assay for HepG-2 cells after 48 h treatment with different concentrations of 5-FU, Ps and combined 5-FU + Ps.

After 2 weeks of HepG-2 cells treatment with different concentrations of 5-FU, Ps extract and combination of 5-FU and Ps extract, it was observed that there was decrease in number of non-adherent colonies in a concentration dependent manner (Fig. 10).

### The effect of 5-FU and/or Ps extract on VEGF, Survivin, BAX and BID gene expression

**Fig. 11:**
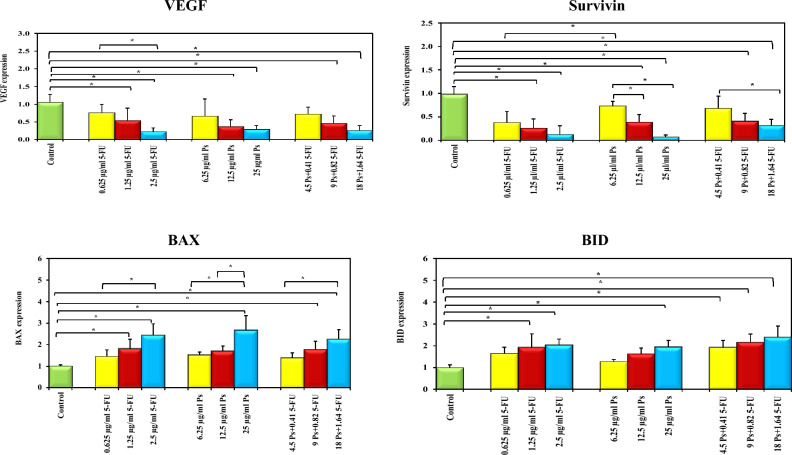
Bar chart representing the effect of 5-FU and/or Ps extract on gene expression of VEGF, survivin, BAX and BID. *: Statistically significant at p ≤ 0.05.

The gene expression of *VEGF* and *survivin* was significantly downregulated in HepG-2 cells treated with different concentrations of 5-FU, Ps extract and combination of 5-FU and Ps extract in a concentration dependent manner compared with untreated cells while, there was insignificant difference between treated groups (Fig. 11). On the other hand, the gene expression of *BAX* and *BID* in all treated groups was significantly upregulated in a concentration dependent manner compared with untreated group while, there was insignificant difference between treated groups (Fig. 11).

### The effect of 5-FU and/or Ps extract on Bcl2 and BAK protein levels

**Fig. 12:**
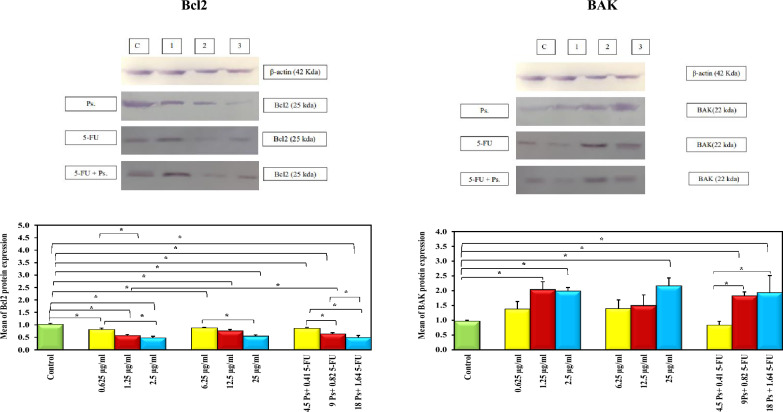
Bar chart representing the effect of 5-FU and/or Ps extract on protein level of Bcl2 and BAK. Where; **C:** Control (untreated HepG-2 cells), **1:** 0.25 IC50, **2:** 0.5 IC50, **3:** IC50. *: Statistically significant at p ≤ 0.05.

The protein levels of Bcl2 in HepG-2 cells treated with different concentrations of 5-FU, Ps extract and combination of 5-FU and Ps extract were significantly decreased in a concentration dependent manner compared to untreated group. On the other hand, the protein levels of BAK were significantly increased in all treated groups in a concentration dependent manner compared to untreated group (Fig. 12).

## Discussion

Sea cucumber, recognized as a medicinal food, has been extensively studied for its anticancer properties^21^. Sea cucumbers contain a wide range of biomolecules, including sulfated polysaccharides, which are powerful chemotherapeutic or chemopreventive agents that have anti-cancer properties by increasing immunity and driving apoptosis in several cancer cell lines^22,23^. The results of this study revealed that 5-FU, sea cucumber Ps extract and combination of 5-FU and Ps extract reduced HepG-2 cells viability in a dose dependent manner. This was similar to the study of Santhanam et al. who reported that aqueous extracts from *Holothuria atra* induced apoptosis in T-47D breast cancer cells^24^. It was found that compounds with an SI > 2 are considered active as anticancer drugs, while those with an SI < 2 may be toxic to normal cells^25^. The results demonstrated that 5-FU exhibited a low selectivity index indicating significant toxicity to normal cells. In contrast, Ps extract and the combination treatment exhibited a high SI, reflecting reduced toxicity to normal cells. Moreover, DNA fragmentation was observed in HepG-2 cells after treatment with various concentrations of 5-FU, sea cucumber Ps extract and combination of 5-FU and Ps extract, indicating DNA breakdown. The DNA fragmentation in cells treated with Ps extract either alone or in combination with 5-FU may be attributed to the presence of phenolic and flavonoids compounds in the Ps extract^26–28^. This was similar to the study of Khaledi, et al., who indicated that treatment of human breast cancer cell line SK-BR-3 cells with Sea cucumber (*H. leucospilota*) extract induced DNA fragmentation^29^.

The results also indicated that Ps extract alone or in combination with 5-FU inhibited the migration of HepG-2 cells. This finding is similar to Yang et al., study which demonstrated that sulfated solysaccharide srom Undaria Pinnatifida could inhibit the proliferation, migration, and invasion and induce apoptosis of ovarian cancer cells by inhibiting the activation of the Hedgehog signaling pathway^30^. Also., Jen et al., indicated that sulphated polysaccharides of Laetiporus sulphureus fruiting bodies markedly inhibited breast cancer cell migration^31^. This finding suggests that Sea cucumber Ps extract can counteract both the locally invasive and metastatic behaviors of tumor cells, indicating its promising anti-metastatic potential for cancer therapy.

Also, 5-FU, Ps extract and combination of 5-FU and Ps extract reduced adherent and non-adherent colony-forming ability of HepG-2 cells. This antitumor and antiproliferative effects of Ps extract against liver cancer cells may be attributed to the presence of phenolic and flavonoid such as gallic acid and kaempferol which have been reported to effectively inhibit soft agar formation in different cancer cell lines^32,33^.

Regarding the effect of sea cucumber Ps extract on angiogenesis, the results indicated that *VEGF* gene expression in cells treated with 5-FU, Ps extract and combination of 5-FU and Ps extract was significantly downregulated by increasing their concentrations compared to untreated cells. The downregulation of *VEGF* by Ps extract either alone or in combination with 5-FU is in accordance to study of Taghdiri et al., who demonstrated that the body-wall extract of the Persian Gulf Sea cucumber (*Holothuria leucospilota*) significantly downregulated the expression of *VEGF* and cyclooxygenase-2 (*COX-2*). Moreover, they found strong relation between expression of *COX-2* and *VEGF* which shows the role of COX-2 in the *VEGF* expression^34^. Previous studies support this, suggesting that COX-2 regulates *VEGF-C* expression through the Prostaglandin E2 receptor (PGE2) pathway and involves two receptors (EP1/EP4) receptors in PGE(2)-mediated VEGF-C production^35,36^. Devanaboyina et al. highlighted that COX-2 is upregulated by NF-κB, contributing to cancer progression. They also reported that Ds-echinoside A from the Sea cucumber *(Pearsonothuria graeffei*) exhibits pro-apoptotic and anti-metastatic effects in HepG2 cells by inhibiting NF-κB-dependent Matrix Metalloproteinase-9 and *VEGF* expression^37^. Additionally, *VEGF* is a primary target gene of hypoxia-inducible factors (HIFs)^38^. Previous studies have shown that gallic acid inhibits VEGF production by reducing HIF-1 expression^39^, and Kaempferol downregulates HIF-1 expression in ovarian carcinomas^40^. Quercetin has been shown to increase HIF-1α degradation through the ubiquitin-dependent pathway^41^.

Additionally, the effect of sea cucumber Ps extract on antiapoptotic markers including Bcl2 and *survivin* and pro-apoptptic markers including *BAX*, BAK and *BID* in HepG-2 cells was assessed. The results indicated that Sea cucumber Ps extract downregulated Bcl2 expression, this finding is consistent with Khaledi et al., who reported that the methanolic extract of Sea cucumber *(Holothuria leucospilota)* inhibited Bcl2 expression in SKBR3 breast carcinoma cells^29^. Similarly, Aslani et al. found that a combination of etoposide and quercetin significantly decreased Bcl2 expression in HepG-2 liver cancer cells^42^. This may be attributed to the downregulation of *VEGF*, leading to reduced Hsp90 levels and subsequent Bcl2 degradation^43^. Also, Ps extract reduced *survivin* expression, previous study reported that the gene expression of *survivin* was significantly reduced in MCF-7 cells treated with Sea cucumber extract^44^. Based on the results of previous studies the downregulation of *survivin* gene expression in HepG-2 cells treated with Ps extract alone or in combination with 5-FU may be attributed to suppression of NF-kB by active compounds in Ps extract including quercetin, gallic acid and kaempferol^45–49^.

On the other hand, sea cucumber Ps extract upregulated *BAX*, BAK and *BID* expression. These findings were in accordance to recent study revealed that Persian Gulf Sea cucumber extracts significantly upregulated *BAX* and *BAK* gene expression in MCF-7 cells^44^. The upregulation of *BAX* and BAK expression in HepG-2 cells treated with Ps extract alone or in combination with 5-FU may be due to hypomethylating effect of quercetin and gallic acid which mediated by downregulation of DNMT1 and DNMT3 which responsible for methylation of *BAX* and *BAK* gene promoters^50–52^. Regarding *BID* expression, it was demonstrated that Phosphoinositide-3 Kinase (PI3K)/protein kinase B (Akt) pathway regulates *BID* expression at the transcriptional level^53^.Thus, the upregulation of *BID* in the current study may be due to the suppression of PI3K/Akt pathway by active components of Ps extract including gallic acid and kaempferol^48,49,53^.

Finally, to the best of our knowledge this is the first study indicated that the sulfated polysaccharides extracted from Sea cucumber have anticancer effect on liver cancer cells which may be mainly through inhibition of PI3K/Akt/NF-kB signaling pathway^37,48,49,53^.

## Conclusion

From this study we concluded that sea cucumber Ps extract can inhibit proliferation of HepG-2 cells through downregulation of *VEGF* and induction of apoptosis in liver cancer cells via downregulation of *survivin* and Bcl2 expression and upregulation of *BAX,* BAK and *BID* expression. Sea cucumber Ps extract may be effective chemotherapeutic agent against HCC.

## Materials and methods

### Collection of sea cucumber

Sea cucumber (*Holothurian atra*) samples were collected from Egyptian Red Sea coastline from National Institute of Oceanography and Fisheries (NIOF) Hurghada station, and the collected sea cucumber samples were transferred into laboratory immediately and maintained at 4 ºC for extraction. Morphological identification of species within the *Holothuria* genus is mainly based on the shape, size and fine details of endodermal ossicles (or sclerites) which are calcified structures that are part of the echinoderm endoskeleton^54^.

### Preparation of sea cucumber sulfated polysaccharides extract

Sea cucumber body were cut into small pieces, digested with 2% neutral protease and soaked in the 70% ethanol. Filtration was used to remove insoluble material, and the digest’s extract was precipitated by adding 30% ethanol (v/v) at 4° C overnight and centrifuging for 10 min at 4500 rpm. The filtrate and precipitate were then separated, and the precipitate was successively washed with ethanol before being dried in an oven to produce the crude polysaccharide extract^55^..

### Characterization of sea cucumber Ps extract

Sea cucumber Ps extract was characterized by fourier transform infrared spectrometer (FT-IR), high performance liquid chromatography (HPLC) and gas chromatography-mass spectrometry (GC–MS)**.**

### Fourier transform infrared spectrometer

To create a fine powder, one milligram of the material was combined with 200 mg of dry potassium bromide. After that, it was compressed and examined in the range of 400–4000 nm using an FT-IR spectrometer (Spectrum One, Perkin Elmer). A RAMII FT-Raman module with a Germanium detector that offered a spectral range of 3600–50 cm^−1^ was connected to a Bruker Model Vertex 70 FT-IR spectrometer to acquire FT-IR data (Bruker, Germany)^56^.

### High-performance liquid chromatography

The phenolic components of sea cucumber Ps extract were detected by Waters 2690 Alliance HPLC (Waters Corp., USA) equipped with column C18 Kromasil 4.6 × 150 mm, 5 μm particle size and a Waters 996 photodiode array detector adjusted at 280 nm. Stock solution of 10 different standards were dissolved in methanol. Each of the standards were filtered using 0.22 μm syringe filter then 10 μl were injected. Extract (38.9 mg/ml) was diluted in methanol and filtered using 0.22 μm syringe filter and 10 μl was injected. With a steady flow rate of 1 ml per minute, the mobile phase consisted of methanol and buffer (0.1 percent phosphoric acid in water) was added. By comparing the relative retention durations of the isolated peaks of the phenolic compounds in the sample with those of the standards, each compound was identified and its concentration (%) was determined using peak area integration^57^.

### Gas chromatography-mass spectrometry

The GC–MS analysis was carried out using gas chromatography–mass spectrometry instrument stands with the following specifications, Instrument: a TRACE GC Ultra Gas Chromatographs (THERMO Scientific Corp., USA), coupled with a thermo mass spectrometer detector (ISQ Single Quadrupole Mass Spectrometer). The GC–MS system was equipped with a TR-5 MS column (30 m × 0.32 mm i.d., 0.25 μm film thickness). Analyses were carried out using helium as carrier gas at a flow rate of 1.0 ml/min and a split ratio of 1:10 using the following temperature program: 60 °C for 1 min; rising at 4.0 °C/min to 240 °C and held for 1 min. The injector and detector were held at 210 °C. Diluted samples (1:10 hexane, v/v) of 1μL of the mixtures were always injected. Using a spectral range of m/z 40–450, mass spectra were obtained by electron ionization (EI) at 70 eV. Using AMDIS software, the chemical components of the essential oil were de-convoluted and identified by its retention indices (relative to n-alkanes C8-C22), mass spectrum matching to authentic standards (when available)., Wiley spectral library collection and NSIT library database^58^.

### Cell culture

The cytotoxic and anticancer effects of sea cucumber Ps extract were determined on HepG-2 cancer and wish normal cells that were divided into four groups according to the treatment regimen received. HepG-2 cancer and wish normal cells were obtained from ATCC, USA. The cells were maintained throughout the study in high glucose RPMI-1640 (Lonza, USA) with 2 mM L-glutamine supplemented with l0% (v/v) heat-inactivated fetal bovine serum (FBS, GIPCO, USA).

### Cytotoxicity assay (MTT assay)

The viability of HepG-2 and wish cells was assessed using 3- (4,5-Dimethylthiazol-2-yl)−2, 5-diphenyltetrazolium bromide (MTT Test)^59^. Briefly, the cells were seeded in a 96-well microtiter plate (1 × 10^5^ cells per well) and allowed to attach to the well bottom for 24 h and treated with different concentrations of 5-FU (0.2, 0.4, 0.8, 1.6, 3.2 and 6.4 µg/ml), Ps extract (20, 40, 80, 160, 200, 300, 400, 500 µg/ml for wish cells and 20, 40, 60, 80, 100, 120, 140 µg/ml for HepG-2 cells) and combination of 5-FU and Ps extract (1 + 10, 2 + 20, 4 + 40, 6 + 80, 8 + 160, 12 + 320 µg/ml for wish cells and 0.1 + 4, 0.2 + 6, 0.4 + 8, 0.8 + 12, 1.6 + 18, 3.2 + 36 µg/ml for HepG-2 cells) in triplicates for 48 h. Then, 20 µL MTT stain (5 mg/ml in PBS) was added to all wells and mixed by agitation at 150 rpm for 5 min. Then, the plates were further incubated for 2 h at the same culture conditions. At the end of the incubation time, purple formazan deposits were formed and dissolved by adding 100 µL DMSO. The absorbance was measured at 570 nm using a microplate reader (Bio-Rad, USA). Then the IC50 and selectivity index (SI) were determined for 5-FU and/or PS.

### Morphological change

HepG-2 cells (2.0 × 10^5^ cells/well) were seeded in 6 well plates and treated with different concentrations of 5-FU (0.625 µg/ml, 1.25 µg/ml, 2.5 µg/ml), Ps extract (6.25 µg/ml, 12.5 µg/ml, 25 µg/ml) and combination of 5-FU and Ps extract (0.41 µg/ml 5-FU + 4.5 µg/ml Ps, 0.82 µg/ml 5-FU + 9 µg/ml Ps, 1.64 µg/ml 5-FU + 18 µg/ml Ps) for 48 h. Then, cells were stained by PI and the morphological changes were examined by using Olympus 1X73 inverted microscope (Olympus, Tokyo, Japan).

### DNA fragmentation assay

DNA fragmentation analysis was determined using the gel electrophoresis technique. 2 × 10^5^ cells were seeded in 6 well plates and treated with different concentrations of 5-FU (0.625 µg/ml, 1.25 µg/ml, 2.5 µg/ml), Ps extract (6.25 µg/ml, 12.5 µg/ml, 25 µg/ml) and combination of 5-FU and Ps extract (0.41 µg/ml 5-FU + 4.5 µg/ml Ps, 0.82 µg/ml 5-FU + 9 µg/ml Ps, 1.64 µg/ml 5-FU + 18 µg/ml Ps) for 48 h. Then, the DNA of untreated (control) and treated cells was extracted using Easy Script kit (TransGen Biotech Co., LTD) as the manufacturer’s instructions. Using nanodrop (Thermo Scientific, USA), the quality and quantity of DNA were determined. For electrophoresis, isolated DNA was run on 2% agarose gel and visualized by Gel Documentation Apparatus (Bio-Rad)^60^.

### Wound healing assay

HepG-2 cells (2.0 × 10^5^ cells/well) were seeded in 6-well tissue culture plates and left overnight in 5% CO2 incubator at 37° C. Then, cells were treated with different concentrations of 5-FU (0.625 µg/ml, 1.25 µg/ml, 2.5 µg/ml), Ps extract (6.25 µg/ml, 12.5 µg/ml, 25 µg/ml) and combination of 5-FU and Ps extract (0.41 µg/ml 5-FU + 4.5 µg/ml Ps, 0.82 µg/ml 5-FU + 9 µg/ml Ps, 1.64 µg/ml 5-FU + 18 µg/ml Ps) for 48 h. Once the cells reached a confluent monolayer, a scrape was carried out in a straight line with a sterile pipette tip on the monolayer. To eliminate cell debris, the detached cells were double washed with PBS. The wound gaps were photographed at regular intervals (0, 24, and 48 h), and the area of cell-free wounds was measured using ImageJ software. Using a phase-contrast microscope, the wound healing was evaluated after 24 h and 48 h in comparison with the control cells^61^.

### Clonogenic assay

HepG-2 cells (1,000 cells/well) were seeded in 6 well plates in complete media. After 24 h, the cells were treated with different concentrations of 5-FU (0.625 µg/ml, 1.25 µg/ml, 2.5 µg/ml), Ps extract (6.25 µg/ml, 12.5 µg/ml, 25 µg/ml) and combination of 5-FU and Ps extract (0.41 µg/ml 5-FU + 4.5 µg/ml Ps, 0.82 µg/ml 5-FU + 9 µg/ml Ps, 1.64 µg/ml 5-FU + 18 µg/ml Ps). Then, plates were incubated at 37 °C for 14 days. After that, the cells were fixed and stained using Crystal Violet (Sigma-Aldrich, USA) at 3% (w/v) in methanol for 5 min at room temperature. The stain was removed and the cells were washed with distilled water^62^. The number of colonies displaying five or more cells was scored under the phase-contrast inverted microscope and the plating efficiency (PE) was calculated using the following formula:

$$PE=\frac{\text{no}.\text{ of colonies formed}}{\text{no}.\text{ of cell seeded}} \times 100$$

### Soft agar assay

HepG-2 cells (4 × 10^4^/ml) was treated with different concentrations of 5-FU (0.625 µg/ml, 1.25 µg/ml, 2.5 µg/ml), Ps extract (6.25 µg/ml, 12.5 µg/ml, 25 µg/ml) and combination of 5-FU and Ps extract (0.41 µg/ml 5-FU + 4.5 µg/ml Ps, 0.82 µg/ml 5-FU + 9 µg/ml Ps, 1.64 µg/ml 5-FU + 18 µg/ml Ps). Then, the cells in dilution of 1:1 were mixed with the 0.6% agarose. 1 ml of the cell agarose mixture was gently added to 0.6% agarose complete medium bottom layer in 6 well plates. The plates were placed horizontally on a flat surface at 4 °C for 15 min to allow the mixture to be solidified. Then, the plates were placed in incubator at 37 °C for a week. After that, 1 ml of feeder layer containing 0.3% agarose/medium/treatment solution was added and allowed to be solidified then plates were placed in a 37 °C incubator after the feeder layer was been solidified. This feeding procedure was repeated weekly until colony formation was observed. Finally, 1 ml of 0.5% of crystal violet was added for 30 min, then it was put in the fridge for 30 min, after 1 h the photos were taken and colonies were counted using a dissecting microscope^63^.

### Quantitative reverse transcription-PCR (qRT-PCR)

The expression of *survivin, BAX, BID* and *VEGF* genes was determined by qRT-PCR**.** HepG-2 cells with density of 2.0 × 10^5^ cells/well were seeded in 6 well plates and treated with different concentrations of 5-FU (0.625 µg/ml, 1.25 µg/ml, 2.5 µg/ml), Ps extract (6.25 µg/ml, 12.5 µg/ml, 25 µg/ml) and combination of 5-FU and Ps extract (0.41 µg/ml 5-FU + 4.5 µg/ml Ps, 0.82 µg/ml 5-FU + 9 µg/ml Ps, 1.64 µg/ml 5-FU + 18 µg/ml Ps) for 48 h. Then, total RNA was extracted from untreated and treated cells using Easy pure RNA kit (TransGen Biotech) according to manufacturer’s protocol. Quantity of the extracted nucleic acid was determined by measuring the absorbance at 260 nm by a NanoDrop™ 2000/2000c spectrophotometer and RNA purity was determined by using the ratio of absorbance values at 260 nm and 280 nm (A260/A280). The complementary DNA (cDNA) was synthesized using Easy Script kit (TransGen Biotech). Finally, gene expression was determined using qPCR Kit (myPOLS Biotech, Konstanz, Germany) according to manufacturer’s protocol by CFX Connect Real-Time PCR (Bio-Rad, USA). PCR primers for target genes and GAPDH housekeeping gene were indicated in Table 1. Using the comparative cycle threshold method (2^-ΔΔCt^), the Real time PCR results were analyzed for calculating fold change of gene expression.

**Table 1:** Primer sequences for target genes.

**Gene**		**Primer sequence**
*BAX*	Forward	5′ TCC CCC CGA GAG GTC TTT T 3′
	Reverse	5′ CGG CCC CAG TTG AAG TTG 3′
*BID*	Forward	5′ CCT TGC TCC GTG ATG TCT TTC3′
	Reverse	5′ TCC GTT CAC TCC ATC CCA TTT 3′
*Survivin*	Forward	5′ CCA CCG CAT CTC TAC ATT C 3′
	Reverse	5′ GTC TGG CTC GTT CTC AGT GC 3′
*VEGF*	Forward	5′TCG AGA CCC TGG TGG ACA TG 3′
	Reverse	5′ TGT TGG ACT CCT CAG TGG GC 3′
*GAPDH*	Forward	5‘AATGGGCAGCCGTTAGGAAA 3’
	Reverse	5‘GCGCCCAATACGACCAAATC 3’

### Western blotting technique

The protein level of Bcl2 and BAK was determined by western blotting assay. HepG-2 cells (2.0 × 10^5^ cells/well) were seeded in 6-well tissue culture plates and treated with different concentrations of 5-FU (0.625 µg/ml, 1.25 µg/ml, 2.5 µg/ml), Ps extract (6.25 µg/ml, 12.5 µg/ml, 25 µg/ml) and combination of 5-FU and Ps extract (0.41 µg/ml 5-FU + 4.5 µg/ml Ps, 0.82 µg/ml 5-FU + 9 µg/ml Ps, 1.64 µg/ml 5-FU + 18 µg/ml Ps) for 48 h. Then, protein was extracted from cells by Protein Ext Mammalian Nuclear and Cytoplasmic Kit (TransGen Biotech) and the concentration of protein was detected by Bradford assay according to manufacturer’s protocol. The cellular protein extracts were separated by sodium dodecyl sulfate–polyacrylamide gel electrophoresis in 15% polyacrylamide gels. Proteins were transferred onto polyvinylidene difluoride membranes by electro-blotting with constant amperage (1 mA/cm2) for 2 h in a wet chamber. After blocking for 1 h by 2% bovine serum albumin in Tris-buffered saline (TBS) at room temperature, membranes were incubated overnight at 4 ^○^C with specific antibodies to Bcl2, Bak and β-actin (Assay Genie, Ireland). After that, membranes were washed 3 times with TBS solution. and probed with their corresponding secondary antibody conjugated to alkaline phosphatase (Assay Genie, Ireland) for 1 h at room temperature. Specific proteins were detected using the enhanced chemiluminescence (ECL) substrate kit (Clarity Western ECL substrate, Bio-Rad, Hercules, California, USA) and the blots were imaged with the C-DiGit LI-COR blot scanner (Bonsai Advanced technologies S.L. Madrid, Spain). Intensities of target protein bands were determined by densitometry and normalized to the intensity of the loading control β-actin protein^64^.

### Statistical analysis of the data

Data were fed to the computer and analyzed using IBM SPSS software package version 20.0*.* (Armonk, NY: IBM Corp) Qualitative data were described using number and percent. The Shapiro–Wilk test was used to verify the normality of distribution. Quantitative data were described using mean ± standard deviation (SD). For normally distributed quantitative variables one-way variance (ANOVA) was used to compare between more than two groups and Post Hoc test (Tukey) for pair wise comparisons. Significance of the obtained results was judged at the 5% level.

## Supplementary Information

Supplementary Information 1.

Supplementary Information 2.

## Data Availability

The datasets used and/or analyzed during the current study are available from the corresponding author on reasonable request.
