# Supplementary material for: Sea cucumber sulfated polysaccharides extract potentiates the anticancer effect of 5- fluorouracil on hepatocellular carcinoma cells
Source: Sci Rep. 2025 Jun 23;15:20255. doi: 10.1038/s41598-025-06496-7 (PMC12185687; doi:10.1038/s41598-025-06496-7)

1. **DNA Fragmentation Images**
2. **5-FU or Ps extract**

M C F1 F2 F3 P1 P2 P3


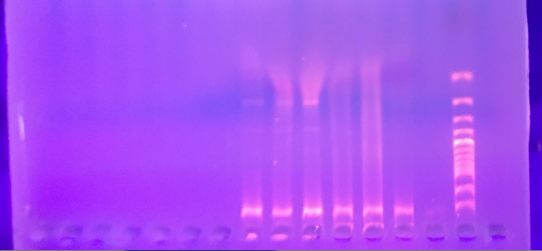


**Figure 1A:** DNA fragmentation in HepG-2 cells treated with different concentrations of 5-FU or Ps extract where, **M**: DNA marker, **C**: control, **F1**: 0.41 µg/ml 5-FU, **F2**: 0.82 µg/ml 5-FU, **F3**: 1.64 µg/ml 5-FU, **P1**: 6.25 µg/ml Ps, **P2**: 12.5 µg/ml Ps, **P3**: 25 µg/ml Ps.

1. **5-FU and Ps in combination**

M C C1 C2 C3


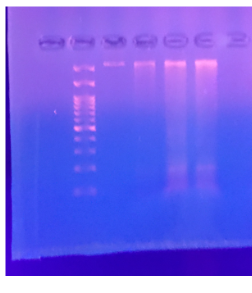


**Figure 1B:** DNA fragmentation in HepG-2 cells treated with different concentrations of 5-FU and Ps extract in combination **where, M:** DNA marker, **C:** control, **C1:** 0.41 µg/ml 5-FU + 4.5 µg/ml Ps, **C2:** 0.82 µg/ml 5-FU + 9 µg/ml Ps, **C3:** 1.64 µg/ml 5-FU +18 µg/ml Ps.

|  |  |  |
| --- | --- | --- |

**2) Western Blot Images**

**A- Effect of 5-FU on BAK protein levels**

**C F1 F2 F3**


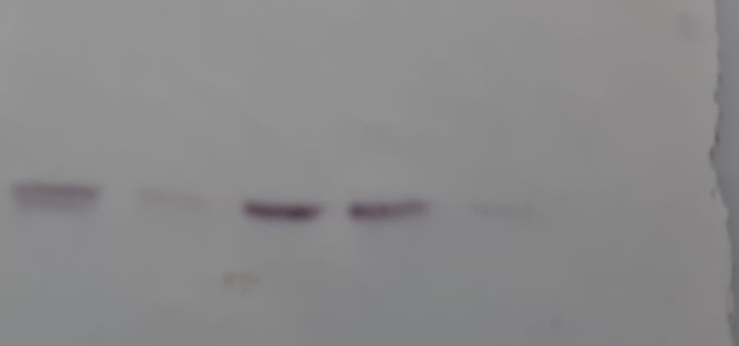


**Figure 2A:** The effect of 5-FU on BAK protein levels in HepG-2 cells where, C: control, **F1:** 0.41 µg/ml 5-FU, **F2**: 0.82 µg/ml 5-FU, **F3**: 1.64 µg/ml 5-FU.

**B- Effect of 5-FU on Bcl2 protein levels**

**C F1 F2 F3**


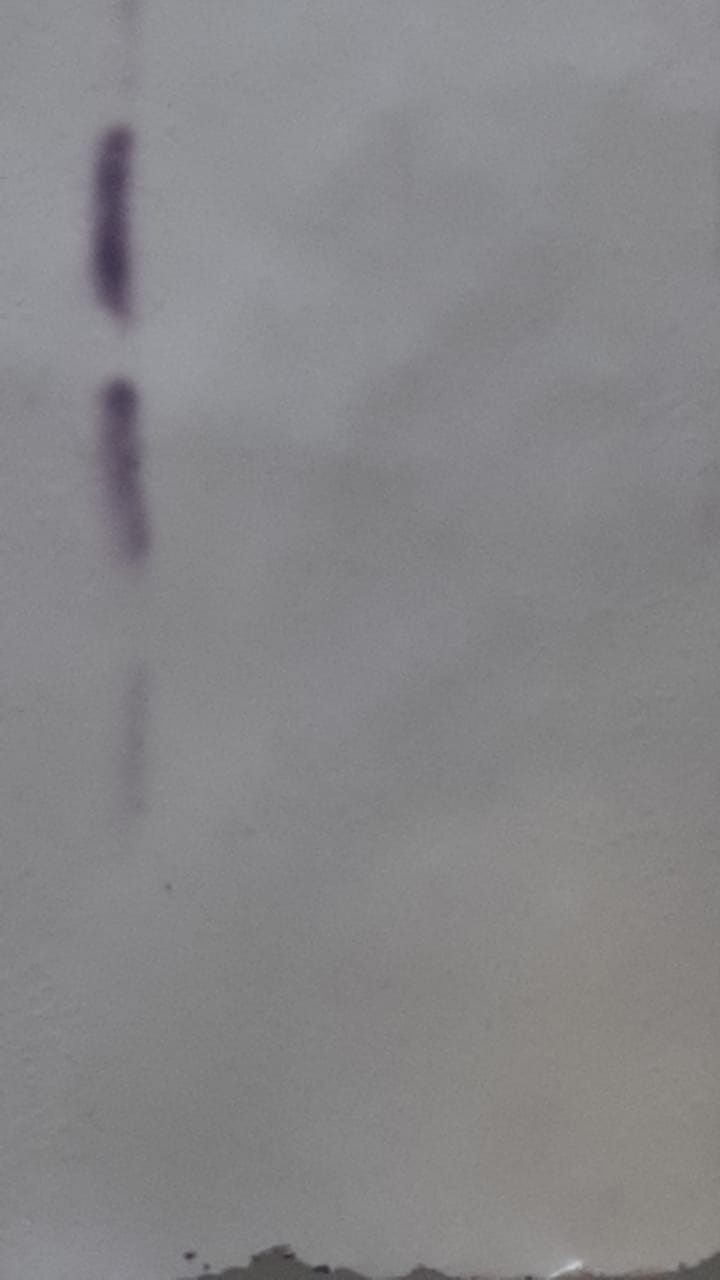


**Figure 2B:** The effect of 5-FU on Bcl2 protein levels in HepG-2 cells where, **C**: control, **F1**: 0.41 µg/ml 5-FU, **F2**: 0.82 µg/ml 5-FU, **F3**: 1.64 µg/ml 5-FU.

1. **Effect of Ps extract on BAK and Bcl2 protein levels**

**BAK Bcl2**

**C P1 P2 P3 C P1 P2 P3**


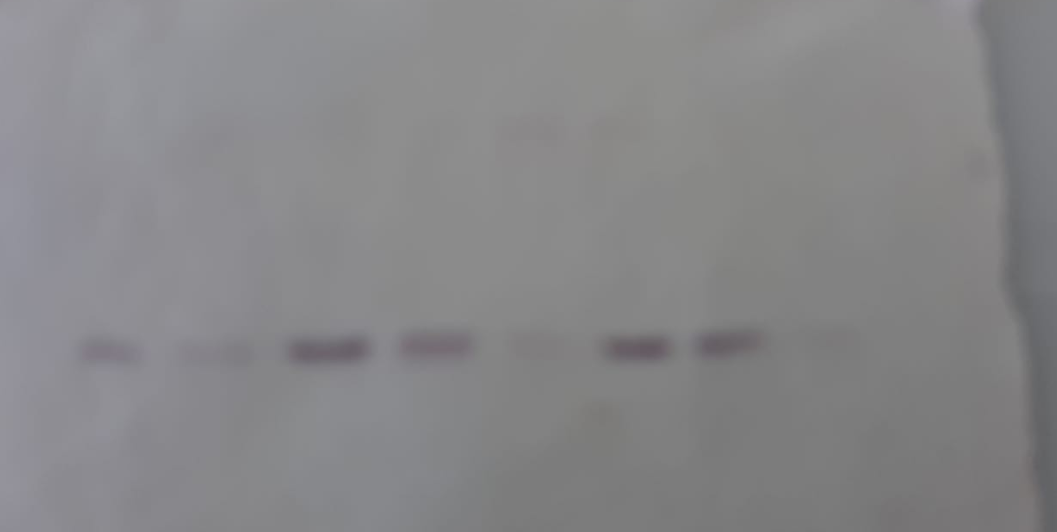


**Figure 2C:** The effect of Ps extract on BAK and Bcl2 protein levels in HepG-2 cells where, **C**: control, **P1:** 6.25 µg/ml Ps, **P2:** 12.5 µg/ml Ps, **P3:** 25 µg/ml Ps.

1. **Effect of 5-FU and Ps extract in combination on BAK and Bcl2 protein levels**

**BAK Bcl2**

**C C1 C2 C3 C C1 C2 C3**


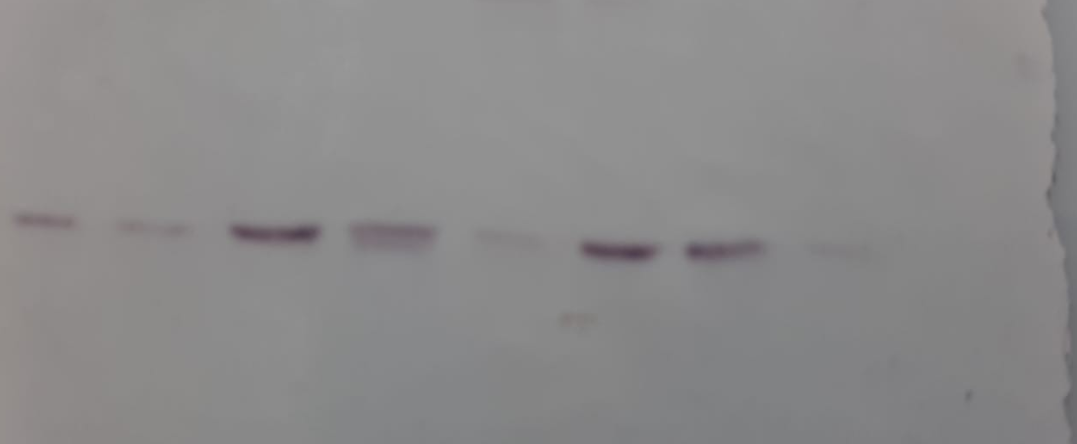


**Figure 2D:** The effect of 5-FU and Ps extract in combination on BAK and Bcl2 protein levels in HepG-2 cells where, **C**: control **C1:** 0.41 µg/ml 5-FU + 4.5 µg/ml Ps, **C2:** 0.82 µg/ml 5-FU + 9 µg/ml Ps, **C3:** 1.64 µg/ml 5-FU +18 µg/ml Ps.

1. **β- actin as a reference protein**


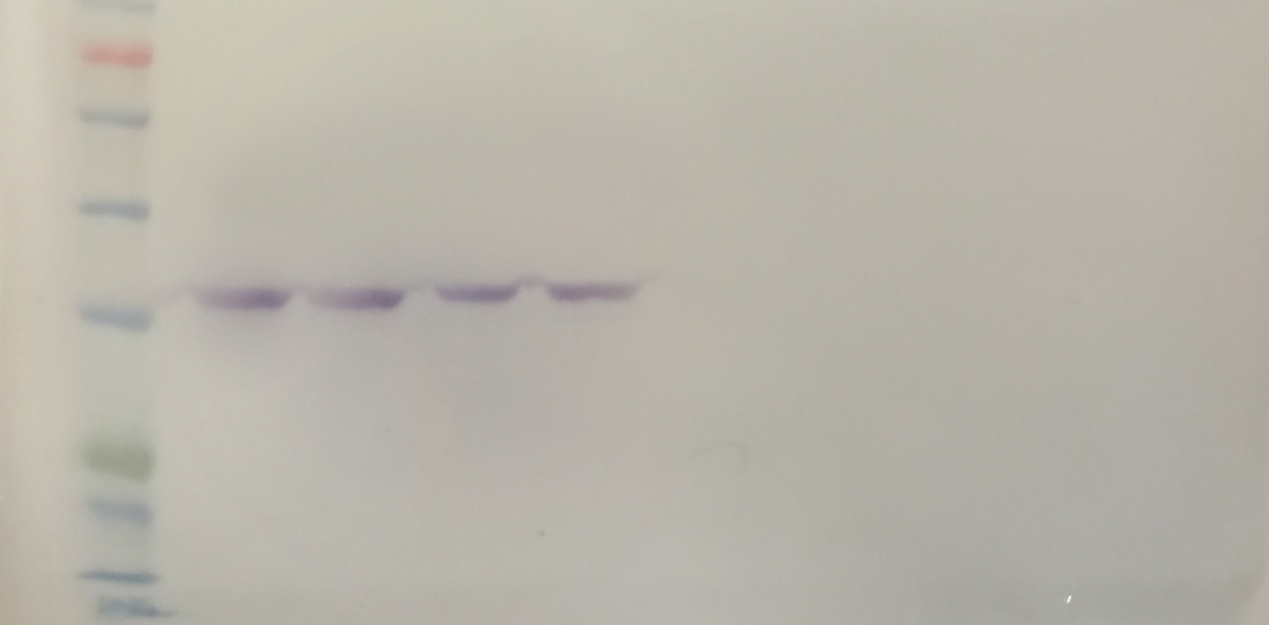

Supplement: Supplementary file 1 — Supplementary Information 1. [file 41598_2025_6496_MOESM1_ESM.docx]
