# Supplementary material for: Sea cucumber sulfated polysaccharides extract potentiates the anticancer effect of 5- fluorouracil on hepatocellular carcinoma cells
Source: Sci Rep. 2025 Jun 23;15:20255. doi: 10.1038/s41598-025-06496-7 (PMC12185687; doi:10.1038/s41598-025-06496-7)
Supplement: Supplementary file 2 — Supplementary Information 2. [file 41598_2025_6496_MOESM2_ESM.docx]

**Table 1: Extract components by IR**

| **Name** | **Product** | **WL in nm** | **No** |
| --- | --- | --- | --- |
| Alcohol | O – H | 3482 | **1** |
| Alkane | C – H | 2935 | **2** |
| 2ndry amine | N – H | 2319 | **3** |
| Isocyanate | N=C=O | 2266 | **4** |
| Alkyne | C ≡ C | 2107 | **5** |
| Amine | N – H | 1632 | **6** |
| Sulfate | S = O | 1414 | **7** |
| Alkane | C – H | 1346 | **8** |
| Fluro compound | C – F | 1199 | **9** |
| Amine | C – N | 1133 | **10** |

**Table 2: Bioactive compounds of Sea cucumber extract**

| **Structural formula** | | **Molecular formula** | | **Nomenclature** | **Area %** | | **RT** | **No** |
| --- | --- | --- | --- | --- | --- | --- | --- | --- |
| 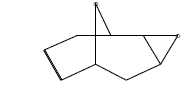 | | C8H1002 | | 3,10-Dioxatricyclo[4.3.1.0 (2,4)] dec-7-ene | 3.13 | | 3.53 | **1** |
| 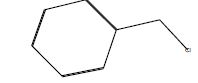 | | C7H7Cl | | Benzene, (Choromethyl) | 2.30 | | 3.97 | **2** |
| 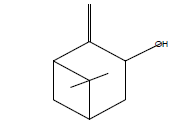 | | C10H16O | | Isopinocarveol | 0.80 | | 4.33 | **3** |
| 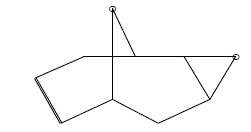 | | C8H1002 | | 3,10-Dioxatricyclo[4.3.1.0 (2,4)] dec-7-ene | 0.92 | | 5.11 | **4** |
| 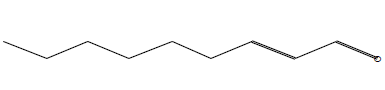 | | C9H16O | | 2-Nonenal, (E) | 1.78 | | 5.64 | **5** |
| 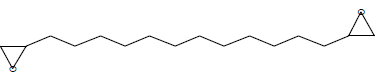 | | C16H3002 | | 1,2-15,16-Diepoxyhexadecane | 0.73 | | 6.73 | **6** |
| 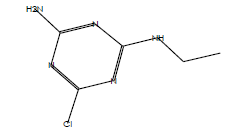 | | C5H8C1N5 | | 1,3,5-Triazine-2,4-diamine,6-Chloro-N-Ethyl | 1.25 | | 7.06 | **7** |
| 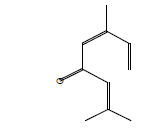 | | C10H140 | | (Z)-2,6-Dimethylocta-2,5,7-trien-4-one | 1.45 | | 7.94 | **8** |
| 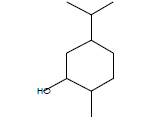 | | C10H140 | | Phenol, 2-methyl-5-(1-methylethyl)- | 10.02 | | 8.15 | **9** |
| 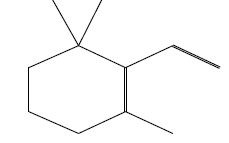 | | C11H18 | | Cyclohexane, 2-ethenyl-1,3,3-trimethyl- | 1.40 | | 8.37 | **10** |
| 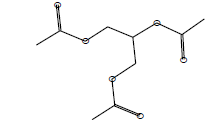 | | C9H14O6 | | 1,2,3-Propanetriol, Triacetate | 0.73 | | 8.98 | **11** |
| 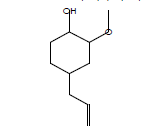 | C10H12O2 | | | Eugenol | 9.19 | 9.19 | | **12** |
| 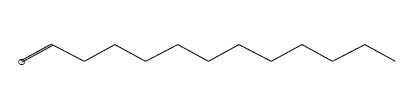 | C12H24O | | | Dodecanal | 10.09 | 10.09 | | **13** |
| 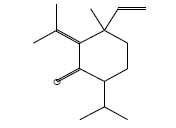 | C15H24O | | | Isoshyobunone | 11.23 | 11.23 | | **14** |
| 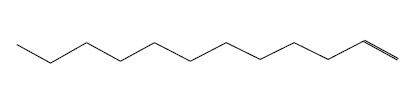 | C12H24 | | | 1-Dodecene | 0.60 | 11.39 | | **15** |
| 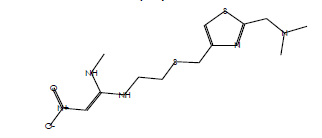 | C12H21N5O2S2 | | | Nizatidine | 11.84 | 11.95 | | **16** |
| 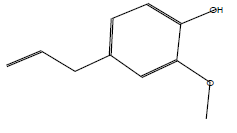 | C10H12O2 | | | Phenol,2-Methoxy-4-(2-Propenyl)- | 0.55 | 12.41 | | **17** |
| 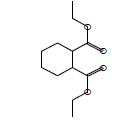 | C12H14O4 | | | Diethyl Phathalate | 3.27 | 13.72 | | **18** |
| 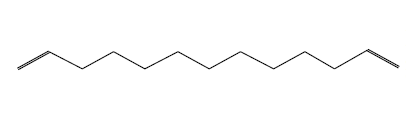 | C13H24 | | | 1,12-Tridecadiene | 0.46 | 14.02 | | **19** |
| 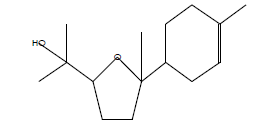 | C15H26O2 | | | Bisabolol oxide B | 6.48 | 14.84 | | **20** |
| 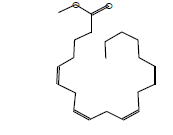 | C21H34O2 | | | 5,8,11,14-Eicosatetraenoic acid, methyl ester A | 1.68 | 15.36 | | **21** |
| 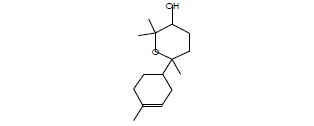 | C15H26O2 | | | 2H-Pyran-3-ol,tetrahydro-2,2,6-trimethyl-6-(4-methyl-3-cyclohexan-1-yl)- | 8.98 | 16.51 | | **22** |
| 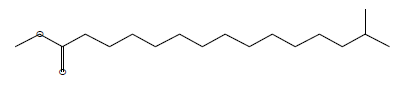 | C17H34O2 | | | Pentadecanoic acid, 14-methyl-methyl ester | 0.84 | 19.57 | | **23** |
| 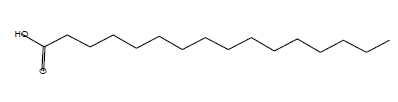 | C16H32O2 | | | Hexadecanoic acid | 7.40 | 20.25 | | **24** |
| 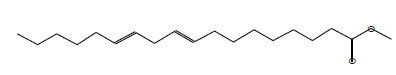 | C19H34O2 | | | 9,12-Octadecadienoic acid(Z,Z)-,methyl ester | 0.41 | 22.23 | | **25** |
| 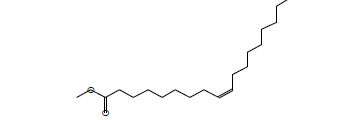 | C19H36O2 | | | 9-Octadecenoic acid (Z)-, methyl ester | 0.52 | 22.33 | | **26** |
| 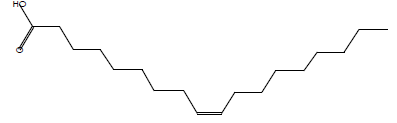 | C18H34O2 | | | Oleic acid | 0.58 | 23.35 | | **27** |
| 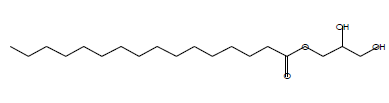 | C19H38O4 | | | Glycerol 1-palmitate | 1.40 | 24.59 | | **28** |
| 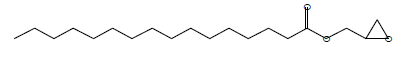 | C19H36O3 | | | Glycidyl palmitate | 1.03 | 25.22 | | **29** |
| 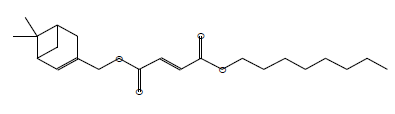 | C22H34O4 | | | Fumaric acid, myrtenyl octyl ester | 0.79 | 25.56 | | **30** |
| 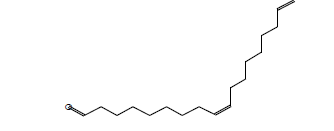 | C18H32O | | 9,17-Octadecadienal, (Z)- | | 3.13 | 27.04 | | **31** |
| 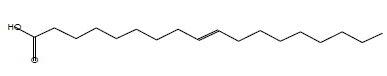 | C18H34O2 | | 9-Octadecenoic acid (Z)- | | 1.35 | 27.65 | | **32** |
